# Safety Assessment of Mefepronic Acid in Sheep

**DOI:** 10.1002/vms3.71236

**Published:** 2026-09-22

**Authors:** Rahmi Canbar, Tugba Melike Parlak, Muhittin Uslu, Enver Yazar

**Affiliations:** ^1^ Department of Pharmacology and Toxicology Faculty of Veterinary Medicine Aksaray University Aksaray Türkiye; ^2^ Department of Pharmacology and Toxicology Faculty of Veterinary Medicine Selcuk University Konya Türkiye; ^3^ Veterinary Laboratory and Veterinary Health Department Sefaatli Vocational School Yozgat Bozok University Yozgat Türkiye

**Keywords:** drug safety, mefepronic acid, oxidative status, sheep

## Abstract

**Background:**

Mefepronic acid is used as a choleretic agent in different animal species. Although mefepronic acid is used extra‐label in sheep, information on its safety in this species is limited. The aim of this study was to determine the effects of mefepronic acid on serum oxidative stress, lipid metabolism and organ function parameters in sheep.

**Methods:**

Mefepronic acid was administered to 10 sheep at a dose of 10 mg/kg (intramuscular, once daily, 3 days). Blood samples were taken at 0 and at 24, 48 and 72 h. 8‐OHdG, MDA, SOD, GPX, CAT, CK‐MB isoenzyme, troponin I, serum ALP, AST, ALT, total bilirubin, BUN, creatinine, cholesterol, triglycerides, LDL and HDL levels were measured.

**Results:**

Mefepronic acid administration resulted in a transient increase in 8‐OHdG levels (*p* < 0.05) and a decrease in CAT levels (*p* < 0.05), a transient increase in CK‐MB isoenzyme levels (*p* < 0.05), and a decrease in BUN, HDL and LDL levels (*p* < 0.05), but no effect on other parameters (*p* > 0.05).

**Conclusion:**

Administration of mefepronic acid to sheep may cause transient changes in oxidative stress parameters and CK‐MB isoenzyme levels. However, these changes were not associated with an increase in troponin I levels or clinically significant myocardial damage. Mefepronic acid did not cause clinically significant adverse effects on liver or kidney function parameters.

## Graphical abstract

This study evaluated the short‐term effects of mefepronic acid in healthy sheep. Mefepronic acid caused transient changes in 8‐OHdG, CAT and CK‐MB isoenzyme levels, but did not produce clinically relevant adverse effects on liver or kidney function parameters.

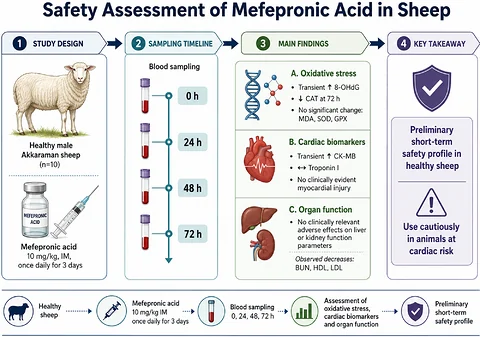

## 1 Introduction

Mefepronic acid (2‐methyl‐2‐phenoxypropionic acid) aids digestion by increasing bile secretion from the liver. Due to its choleretic effect, it is used to treat diseases such as liver failure, icterus, anorexia, tympani and meteorismus. After intramuscular (IM) administration, the drug is rapidly absorbed and eliminated unchanged in the urine and faeces. Mefepronic acid can be used in pregnant and lactating animals and does not cause significant side effects in target species, which include horses, cattle, goats, pigs and dogs. The recommended dose is 10 mg/kg (IM, once daily [SID]), for 1–3 days (HBS 2025; Tras 2024; Yazar 2018). Studies in rats have reported that mefepronic acid has a limited protective effect on hepatic steatosis but is beneficial for treatment (Gumuş et al. 2013). Off‐label use in sheep for non‐choleretic purposes has been reported in the literature. No effects on fertility were reported after combined progesterone and mefepronic acid (10 mg/kg, IM) injections in sheep in the early and late postpartum periods outside the breeding season (Kutlu et al. 2023), or after mefepronic acid (10 mg/kg, IM) administration on Day 9 after mating (Kutlu and Akbulut 2025). It has been suggested that administering mefepronic acid in combination with triclabendazole and levamisole to sheep with fasciolosis could help correct the associated severe metabolic liver dysfunction and negative energy balance (Yuksek et al. 2013). Furthermore, use of mefepronic acid alone or in combination with propylene glycol has been reported in the treatment of pregnancy toxaemia and fatty liver syndrome in sheep (Da Silva et al. 2016). However, overall information on the drug's use in sheep is limited, and there is no information on its safety or clinical use.

Free oxygen radical species (ROS) are unstable structures produced in cells that can damage various biological structures. To counter this damage, ROS are converted into harmless structures within the body by enzymatic processes (superoxide dismutase [SOD], catalase [CAT], glutathione peroxidase [GPX], etc.) and non‐enzymatic substances (glutathione, vitamin C, vitamin E, vitamin A, etc.) (Tabakoglu and Durgut 2013). A specific example is the mitochondrial respiratory chain, in which an oxygen molecule gains an electron and transforms into a superoxide radical. SOD enzyme combines two superoxide radicals with two hydrogen ions to form hydrogen peroxide (H_2_O_2_). Then, hydrogen peroxide is transformed into water and molecular oxygen by GPX and CAT. In the living organisms, an equilibrium presents between oxidants and antioxidants. The disruption of this balance in favour of oxidants is stated as oxidative stress. The unneutralized ROS attacks cellular structures, such as lipids, proteins and DNA leading to the degradation of these macromolecules. Cell membrane lipids are highly susceptible to ROS, and this degradation is defined as lipid peroxidation (Yazar and Tras 2002). This process produces malondialdehyde (MDA), and the measured MDA can be assessed by the level of oxidative stress in a biological system (Sezer and Keskin 2014). MDA has cytotoxic, mutagenic and carcinogenic effects, and inhibits the activity of enzymes involved in defending cells against further oxidative stress (Całyniuk et al. 2016). 8‐hydroxy‐2‐deoxyguanosine (8‐OHdG) is produced during the repair of DNA damage caused by oxidative stress and is considered the most common biomarker of DNA damage (Di Minno et al. 2016). 8‐OHdG also has a mutagenic effect (Pilger and Rüdiger 2006).

While drugs can have beneficial effects on living organisms, undesirable side effects can also occur at administered doses. Side effects of drugs can be observed clinically or understood through parameters measured in blood, serum or plasma. Physiological or biochemical values obtained through these tests can indicate damage to certain systems or organs (Doğan 2011; Kaya and Unsal 2002). In sheep, measurement of serum creatine kinase (CK‐MB) isoenzyme and troponin I levels have been used to determine cardiac damage (Coskun et al. 2019b; Ider et al. 2020). Meanwhile, serum levels of alkaline phosphatase (ALP), aspartate aminotransferase (AST), alanine aminotransferase (ALT) and total bilirubin (TB) provide information about liver and bile duct functions (Turgut 2000a); triglyceride, cholesterol, high‐density lipoprotein (HDL) and low‐density lipoprotein (LDL) levels reflect lipid metabolism (Turgut 2000b). Finally, creatinine and blood urea nitrogen levels provide information about kidney function (Kerr 2002).

There is limited information on the use of mefepronic acid in sheep. While tilmicosin is safe for use in cattle, it has been reported to cause mortality in goats (Yazar 2018). Anthelmintic and ectoparasitic ivermectin is generally safe for dogs but it causes toxicity and mortality in Collies (Mealey et al. 2001). Given that off‐label use of mefepronic acid can reduce lipid peroxidation in liver hepatocytes (Mecitoglu et al. 2017) and, as with other drugs, affects organ function parameters (Doğan 2011; Kaya and Unsal 2002), we hypothesized that it may affect serum markers of oxidative status, lipid metabolism and organ function in sheep.

The purpose of this research was to determine the effects of mefepronic acid administered to sheep at the extra‐label used dose (10 mg/kg, SID, IM, 3 days) on markers of oxidative status (8‐OHdG, MDA, SOD, GPX, CAT), organ function (troponin I, CK‐MB isoenzyme, ALP, ALT, AST, TB, BUN, creatinine) and lipid metabolism (cholesterol, triglyceride, HDL, LDL).

## 2 Materials and Methods

### 2.1 Animal Experiments

The research procedure was approved by the Selcuk University Faculty of Veterinary Medicine Experimental Animal Production and Research Center Ethics Committee (SUVDAMEK, No: 2025/78).

The study was conducted on 10 male Akkaraman sheep (8–12 months old, 45–55 kg). Sheep were supplied with food and water ad libitum. All animals were housed under the same environmental conditions. Mefepronic acid (Hepagen Inj Sol, Fatro, Italy) was administered to the animals at a dose of 10 mg/kg (IM, SID, 3 days) into the gluteal muscles. Blood samples were obtained before the treatment (Day 0) and at 24, 48 and 72 h after the first dose.

### 2.2 Laboratory Procedures

The obtained blood samples were centrifuged at 4000 × *g* for 10 min. Serum levels of 8‐OHdG, MDA, SOD, GPX, CAT, troponin I and CK‐MB isoenzymes were determined using commercial ELISA kits specific to sheep (BT‐LAB, Bioassay Technology Laboratory, Zhejiang, China) and an ELISA reader (MWGt Lambda Scan 200, Bio‐Tec Instruments, Winooski, VT, USA). Serum AST, ALT, ALP, TB, BUN, creatinine, cholesterol, triglyceride, HDL, LDL and levels were determined using an autoanalyzer (BT 3500I, Instrumentation Laboratory Company, Milan, Italy).

### 2.3 Statistical Analysis

Study results are described as mean ± standard error (SE). In the study conducted, data were normally tested using the Shapiro–Wilk test. While BUN, AST, ALT, ALP, creatinine, cholesterol, TB, triglycerides, LDL, troponin I, SOD, MDA and CAT parameters were found to be normally distributed, HDL, CK‐MB isoenzyme, 8‐OHdG and GPX parameters were found to be non‐normally distributed. Data that were not normally distributed were transferred to log10. All data were evaluated using Repeated Measures ANOVA and Bonferroni post‐hoc tests (SPSS 29.0). A *p*‐value < 0.05 was accepted as the statistical significance level.

## 3 Results

Mefepronic acid administration in sheep was observed to cause no clinical adverse effects (fever, shivering, salivation, etc.). Effect of mefepronic acid on the serum oxidative status values are shown in Table 1. A transient increase in 8‐OHdG levels (*p* < 0.05; 24 h) and a decrease in CAT levels (72 h; *p* < 0.05) were observed. It also caused non‐significant fluctuations in MDA, SOD and GPX levels (*p* > 0.05).

**TABLE 1:** Effect of mefepronic acid on the serum oxidative status values in sheep (mean ± SE).

Parameters	0 h	24 h	48 h	72 h
8‐OHdG ng/mL	4.86 ± 0.51^b^	7.37 ± 0.42^a^	6.92 ± 0.52^ab^	6.03 ± 0.34^ab^
MDA nmol/L	5.77 ± 0.66	5.34 ± 0.40	5.06 ± 0.29	4.80 ± 0.49
SOD ng/mL	70.42 ± 7.97	61.55 ± 4.89	59.87 ± 3.26	62.45 ± 3.71
GPX nU/mL	638.82 ± 123.17	466.33 ± 34.03	572.01 ± 26.45	571.22 ± 57.96
CAT ng/mL	4.22 ± 0.42^a^	3.22 ± 0.28^ab^	3.47 ± 0.28^ab^	3.02 ± 0.23^b^

*Note*: 8‐OHdG and GPX values were log10‐transformed before statistical analysis.

Abbreviations: 8‐OHdG, 8‐hydroxy‐2‐deoxyguanosine; CAT, catalase; GPX, glutathione peroxidase; MDA, malondialdehyde; SOD, superoxide dismutase.

^a,b^ Different letters in the same line are statistically significant (*p* < 0.05).

The effects of mefepronic acid on CK‐MB isoenzyme and troponin I levels in sheep are presented in Table 2. A temporary increase in CK‐MB isoenzyme levels (*p* < 0.05; 24 h) was observed.

**TABLE 2:** Effect of mefepronic acid on cardiac damage values in sheep (mean ± SE).

Parameters	0 h	24 h	48 h	72 h
CK‐MBiso ng/mL	0.62 ± 0.07^b^	1.01 ± 0.11^a^	0.57 ± 0.04^ab^	0.70 ± 0.07^ab^
Troponin I ng/mL	119.50 ± 13.83	89.33 ± 12.94	123.03 ± 14.24	129.87 ± 15.47

*Note*: CK‐MB isoenzyme values were log10‐transformed before statistical analysis.

Abbreviation: CK‐MBiso, creatine kinase‐MB isoenzyme.

^a,b^ Different letters in the same row are statistically significant (*p* < 0.05).

The effects of mefepronic acid on serum organ function parameters and lipid metabolism values in sheep are presented in Table 3. Mefepronic acid caused a decrease (*p* < 0.05) in BUN (72 h), HDL (72 h) and LDL (24 and 72 h) levels. The drug also caused non‐significant (*p* > 0.05) decreases in AST, ALP, TB and cholesterol values​, and fluctuations in other parameters.

**TABLE 3:** Effect of mefepronic acid on serum organ function and lipid metabolism values in sheep (mean ± SE).

Parameters	0 h	24 h	48 h	72 h
ALP U/L	460.71 ± 60.18	420.54 ± 42.66	369.53 ± 35.68	355.12 ± 39.74
ALT U/L	15.25 ± 2.22	13.74 ± 1.52	15.52 ± 2.40	15.34 ± 2.72
AST U/L	73.78 ± 8.41	69.13 ± 4.38	71.49 ± 5.25	70.29 ± 4.69
TB mg/Dl	0.35 ± 0.07	0.32 ± 0.04	0.31 ± 0.06	0.24 ± 0.03
BUN mg/dL	11.31 ± 0.58^ab^	10.19 ± 0.41^ab^	10.73 ± 0.66^a^	9.01 ± 0.65^b^
Creatinine mg/dL	0.59 ± 0.08	0.51 ± 0.07	0.48 ± 0.07	0.52 ± 0.05
Cholesterol mg/dL	30.28 ± 3.28	27.74 ± 1.26	23.26 ± 2.64	23.15 ± 1.59
Triglyceride mg/dL	9.15 ± 2.15	13.27 ± 2.33	6.15 ± 1.66	7.70 ± 1.56
HDL mg/dL	33.20 ± 1.51^a^	33.25 ± 1.21^a^	30.83 ± 1.57^ab^	29.78 ± 1.18^b^
LDL mg/dL	27.50 ± 2.32^a^	24.70 ± 2.01^b^	21.40 ± 2.56^abc^	19.00 ± 2.45^c^

*Note*: HDL values were log10‐transformed before statistical analysis.

Abbreviations: ALP, alkaline phosphatase; ALT, alanine aminotransferase; AST, aspartate aminotransferase; BUN, blood urea nitrogen; HDL, high‐density lipoprotein; LDL, low‐density lipoprotein; TB, total bilirubin.

^a,b,c^ Different letters in the same row are statistically significant (*p* < 0.05).

## 4 Discussion

Mefepronic acid increases liver and pancreatic secretions, ensuring the efficient functioning of digestive organs. Due to its choleretic effect in target species, it is used in the treatment of diseases such as liver failure, icterus, anorexia, tympani and meteorismus (HBS 2025; Tras 2024; Yazar 2018).

In this study, mefepronic acid caused a transient increase in serum 8‐OHdG levels (*p* < 0.05) and a decrease in CAT levels (*p* < 0.05) in sheep (Table 1). No change was detected in SOD, MDA or GPX levels (*p* > 0.05, Table 1). 8‐OHdG is produced during the repair of DNA damage caused by oxidative stress (Di Minno et al. 2016; Pilger and Rüdiger 2006), and its levels have been investigated for several drugs used in sheep (Canbar et al. 2023; Coskun et al. 2019a; Ider et al. 2020). Possibly due to mefepronic acid's reduction of lipid peroxidation in hepatocytes or its choleretic effect, it and ammonium molybdate may be effective for copper poisoning in sheep (Mecitoglu et al. 2017). SOD acts by converting ROS into H_2_O_2_ in the antioxidant system (Fridovich 1995). The GPX family reduces H_2_O_2_ or fatty acid hydroperoxides, which are harmful to cells (Arthur 2000). CAT has a similar effect, converting the H_2_O_2_ produced by SOD into molecular oxygen and water (Yazar and Tras 2002). MDA levels, formed during lipid peroxidation (Esterbauer et al. 1991) are a biomarker of oxidative stress (Sezer and Keskin 2014). In healthy sheep, mefepronic acid administration caused a transient increase in 8‐OHdG levels, but since this was not accompanied by significant changes in MDA, SOD or GPX levels, it can be stated that it cannot be directly interpreted as DNA damage.

In the current research, mefepronic acid caused a temporary increase (*p* < 0.05; 24 h) in CK‐MB isoenzyme levels, while troponin I (*p* > 0.05) levels did not change, which are cardiac damage markers (Table 2). CK‐MB isoenzyme and troponin I levels have been frequently investigated in the drug toxicity studies in sheep (Corum et al. 2015, 2016; Coskun et al. 2018). When blood flow is limited in the coronary arteries, which provide oxygen to the heart muscle, myocardial infarction develops. Hence, necrosis and cell death occur in the myocytes (Stark et al. 2025). Disruption of cell membranes due to hypoxia or other injuries causes CK to be released from the cellular cytosol into the systemic circulation. Hence, increases of serum CK concentrations are accepted as a sensitive but nonspecific marker for cardiac damage (Cabaniss 1990). In the cardiac muscle, the CK‐MM levels constitute 70%–85% of the total CK activity, and nearly all the remaining activity forms CK‐MBiso. However, it is produced in very small amounts in the uterus, small intestine, diaphragm, tongue and prostate (Robinson and Christenson 1999). Although the CK‐MB isoenzyme is frequently used as a biomarker of cardiac damage, some research has questioned whether it is found only in the heart and only released in cases of myocardial infarction. It is also unclear whether the level and time of serum CK‐MB elevation reflect the extent of cardiac damage (Guzy 1977). However, CK‐MB isoenzyme levels are used in cardiac screening with other enzymes, such as cardiac troponin I, troponin T, lactate dehydrogenase (LDH) and myoglobin (Robinson and Christenson 1999). In the past, CK‐MB has been accepted as the reference diagnostic markers of acute myocardial injury, but its use is restricted due to its lack of cardiac specificity and rapid clearance from the circulating blood. Elevation of cardiac troponin has been recommended as the determination of myocardial infarction (Al‐Otaiby et al. 2011). Troponins were first discovered in 1965, but a reliable immunological analysis method was developed in the 1990s. Cellular content, including troponins, can be identified in the blood after cell death. Immunologically, it is probable to differentiate cardiac troponin from skeletal troponin, increasing the specificity of assays. Before troponin, biomarkers such as AST, LDH and CK were used to detect myocardial injury. These were discontinued due to their poor specificity for cardiac muscle. Next‐generation biomarkers, such as CK‐MB and LDH 1–2, are reported to be more specific to cardiac muscle, but still yield unacceptably high levels of false‐positive results (Stark et al. 2025). When the findings of the current study are evaluated against the literature, the transient increase in CK‐MB isoenzyme levels without changes in troponin I suggests that no clinically significant myocardial damage was observed during the short study period. Furthermore, given the small sample size (*n* = 10), short observation period and the fact that the study was conducted on healthy sheep, these findings should be interpreted cautiously and not generalized to overall cardiac safety. Therefore, particular caution may be required in animals with pre‐existing heart disease or other relevant risk factors.

Mefepronic acid caused a decrease (*p* < 0.05; 72 h) in BUN levels within reference values (Bulbul 2013) (Table 3). BUN and creatinine clearance are frequently used in screening tests for kidney function (Canbar and İçigen 2025). All creatinine is filtered by the glomeruli and excreted in the urine. Serum creatinine levels provide specific information on the glomerular filtration rate (Miller and Inker 2020). Although there is a relationship between BUN and creatinine production and excretion, BUN values must be supplemented with creatinine clearance for a reliable assessment of kidney function. Creatinine clearance is a more accurate indicator of function than BUN in all stages of renal failure, because BUN levels are more strongly influenced by diet and physiological variables (Hosten 1990). BUN levels can also be affected by renal tubular reabsorption (Otto 2017). Although a decrease in BUN values was observed in the study, this occurred alongside a change in reference values and a lack of change in creatinine values. This suggests that mefepronic acid does not cause abnormal changes in kidney function but should be used with caution in renal disease risk groups.

Mefepronic acid caused a decrease in LDL and HDL levels (*p* < 0.05). Mefepronic acid caused non‐significant (*p* > 0.05) decreases in AST, ALP, TB and cholesterol values, and fluctuations were determined in other parameters as well (*p* > 0.05, Table 3). It has been reported that mefepronic acid can be used alone or in combination with propylene glycol in the treatment of fatty liver and associated pregnancy toxemia (Da Silva et al. 2016). Prepartum and postpartum administration of mefepronic acid to overweight and normal‐weight cows has been reported to have no effect on serum lipid parameters (Aparicio‐Cecilio et al. 2012) and may be beneficial in improving liver function in postpartum dairy cows (Rizzo et al. 2014). It has also been reported that mefepronic acid can be used as a supportive treatment in dogs with hepatopathy (Quintavalla et al. 2021). When the literature and current study data are interpreted together, it shows that mefepronic acid does not cause clinically significant adverse effects on liver or kidney function parameters in healthy sheep. This effect may be more pronounced in sick animals or may be used for preventive purposes during birthing.

This study was conducted using a repeated measures and self‐controlled design, with each animal compared to its baseline value. This approach helped reduce the impact of inter‐individual biological differences and allowed for the evaluation of time‐dependent changes following mefepronic acid administration. However, the absence of a separate control group limits the complete separation of drug‐related effects from spontaneous or time‐dependent biological variability. Therefore, the findings should be interpreted within the limitations of this experimental design.

Furthermore, the study data was evaluated in a relatively small, short‐term observation period. Therefore, the current results should be considered as preliminary data obtained from healthy sheep. Long‐term studies with larger sample sizes are needed in future research.

## 5 Conclusions

In conclusion, the extra‐label use of mefepronic acid may temporarily cause oxidative stress in healthy sheep and should be used with caution in animals with heart disease. Administration of mefepronic acid to sheep may cause transient changes in oxidative stress parameters and CK‐MB isoenzyme levels. However, the absence of a concomitant increase in troponin I suggests that it does not indicate clinically significant myocardial damage. Nevertheless, it should be used cautiously in animals at risk. When the study data were evaluated, no clinically significant adverse effects on liver or kidney function parameters were observed. These effects may be more pronounced in sick animals. The study data have been obtained from healthy animals. Future studies should be conducted in different breeds, sick or pregnant animals to evaluate the clinical implications of these effects.

## Author Contributions

All authors contributed to the study conception and design. **Rahmi Canbar**: writing – review and editing, methodology, conceptualization, project administration, formal analysis. **Tugba Melike Parlak**: writing – review and editing, visualization, supervision, data curation, original draft, resources, investigation. **Muhittin Uslu**: writing – review and editing, supervision, original draft, resources, investigation. **Enver Yazar**: investigation methodology, conceptualization, project administration, data curation, formal analysis.

## Funding

This study was financed by Scientific Research Projects Coordinatorship of Selcuk University (SUBAPK 25401129).

## Ethics Statement

The research procedure was approved by the Selcuk University Faculty of Veterinary Medicine Experimental Animal Production and Research Center Ethics Committee (SUVDAMEK, no: 2025/78).

## Conflicts of Interest

The authors declare no conflicts of interest.

## Data Availability

The data that support the findings of this study are available on request from the corresponding author. The data are not publicly available due to privacy or ethical restrictions
